# Hemostatic chemical constituents from natural medicine *Toddalia asiatica* root bark by LC-ESI Q-TOF MS^E^

**DOI:** 10.1186/s13065-017-0283-3

**Published:** 2017-06-15

**Authors:** Xiaoyan Zhang, Wenbo Sun, Zhou Yang, Yan Liang, Wei Zhou, Lei Tang

**Affiliations:** 1 School of Basic Medical Sciences, School of Pharmacy, Guizhou Medical University, Guiyang, 550025 China; 2 Department of Resources Development, Shanghai Standard Biotech Co., Ltd., Shanghai, 201203 China

**Keywords:** Q-TOF, MS^E^, *Toddalia asiatica* root bark, Qualitative analysis, Chemical database

## Abstract

**Background:**

*Toddalia asiatica* root bark as an effective hemostatic natural medicine or Chinese materia medica was applied in China for long history, its complex drug action mechanisms and unclear substance basis have been constraining the development of this drug.

**Results:**

An intelligentized strategy by LC-ESI Q-TOF MS^E^ was presented in this study for rapid identification of hemostatic chemical constituents from this natural medicine. Chromatographic separation was performed on a C18 column (150 mm × 2.1 mm, 1.8 μm), the MS^E^ data in both negative and positive ion modes were acquired to record the high-accuracy MS and MS/MS data of all precursor ions. To reduce the false positive identifications, structural confirmation was conducted by comparison with the isolated reference standards (t_R_ and MS, MS/MS data) or matching with natural product databases. Bioassay-guided fractionation of the extract of *T. asiatica* root bark was also carried out.

**Conclusions:**

As a consequence, 31 natural compounds in *T. asiatica* root bark got putatively characterized. There were four main coumarins, isopimpinellin (Cp.23), pimpinellin (Cp.24), coumurrayin (Cp.30) and phellopterin (Cp.34) isolated and identified from *T. asiatica* root bark. The present study provided candidate strategy that helps to effectively identify the primary natural compounds of TCM or other complex natural medicines, and then promote development and application of natural medicines and their medicinal resources.

## Background

Chinese materia medica as an integral part of traditional Chinese medicine (TCM) system is constantly being applied and validated for curing human diseases and maintaining health over 5000 years of Chinese history and civilization, Chinese materia medica and TCM system have achieved great success and accumulated invaluable experiences in the clinic. Therapeutical effects of TCMs around the world for curing a certain intractable disease are usually from whole outcomes of multiple constituents in the clinic, no matter what single TCM or compound prescription is applied by the patients. Unfortunately, according to new drug criterion of the modern western country, some single compounds extracted and separated from a TCM sometimes can’t show definite pharmacological effects in vivo and in vitro while their extracts of original TCMs have obvious therapeutical outcomes either in lab or in the clinic. To finish a suit of GLP, GCP including Phase I–IV, drug register in the local drug administration department and GMP for developing a new drug (natural medicines also need to add GAP in addition to the above) will invest several dozen millions of research cost, undergo dozens of year. Complex drug action mechanisms and drug substances of Chinese materia medica have been worldwide problems until today, unclear effective compounds of Chinese materia medica is a great gulf in the way of TCMs modernization.

LC-ESI Q-TOF MS^E^ is a very efficient method under high resolution of Time-of-Fly (TOF) MS and MS^E^ module, helping accurately record, qualify and quantify parent ions, daughter ions. It is a very efficient way to help rapidly elucidate complex drug substances such as TCMs, and then further explain the pharmacological mechanisms [1–3]. Of course, professional natural medicine databases are essential, Reaxys database (https://www.reaxys.com/) as a new commercial product of Elsevier company is very useful to chemical scholars, as a combination of Beilstein database, Patent database and Gmelin database, it covers more than 20 million compounds and 30 million chemical reactions, developes many unique searching items such as nature products, reaction rather than SciFinder. It is currently the most comprehensive and largest database of nature products. SIOC chemical database (http://www.organchem.csdb.cn/), CAS developed by Shanghai Institute of Organic Chemistry (SIOC) is the general informational system for chemical and chemical engineering research and development. It consists of more than 20 different chemical databases, such as compound structure database, Chinese traditional medicine, chemical technical information database [4].

*Toddalia asiatica* root bark (*T. asiatica*), or Feilong Zhangxue in Chinese name as Miao minority medicines is dried root bark of *Toddalia asiatica* (L.) Lam. from *Toddalia* genus, Rutaceae family. Its original plant distributes in mountains, valley areas of Guizhou, Guangxi, Yunnan province in China. *T. asiatica* obtains dispelling pathogenic wind and pain, eliminating stasis and hemostasis, subduing swelling and detoxicating in traditional Chinese medicine system. This Miao herbal medicines is always applied in the clinic for treatment of rheumatic arthralgia, stomach pain, hemorrhage, gingival bleeding, etc., which has been recorded in ‘Quality standards of Chinese medicinal materials and ethnomedicines in Guizhou (2013 edition)’, standard number: DB52/YC059-2003 [5]. Until now, almost reported literature were focused on whole root, stem parts of *T. asiatica* [6], but ignoring the root bark part which is main medicinal part based on traditional ethnomedicine [7]. So it’s necessary to scientifically explore the chemical constituents in *T. asiatica* root bark. Therefore, we will apply such high-resolution tandem MS, assisted with natural product databases to systematically explain the hemostatic chemical constituents from *T. asiatica* root bark, the finding in this study will help us to deeply realize therapeutical effect of *T. asiatica* and promote its modernization and new drug development.

## Experimental

### Materials and reagents

All *T. asiatica* were collected from Huaxi district, Guiyang city in Sept. 2013 and were identified by Professor Deyuan Chen from School of Pharmacy at Guiyang College of traditional Chinese medicine. A voucher specimen was stored at Standard Library of traditional Chinese medicine and ethnic medicine, School of Pharmacy, Guizhou Medical University. Some dirty root bark were rinsed with water to remove soil particles and were then sundried. *T. asiatica* samples were ground into powder of the homogenous 24 mesh before the experiment. Acetonitrile (HPLC-pure, Tedia, USA), formic acid (HPLC-pure, Aladdin, China), Methanol (HPLC-pure, Sinopharm, China), Ultra-pure water purified with a Milli-Q water purification system (USA). All other chemicals were of analytical grade.

### Extraction and isolation

**Fig. 1:**
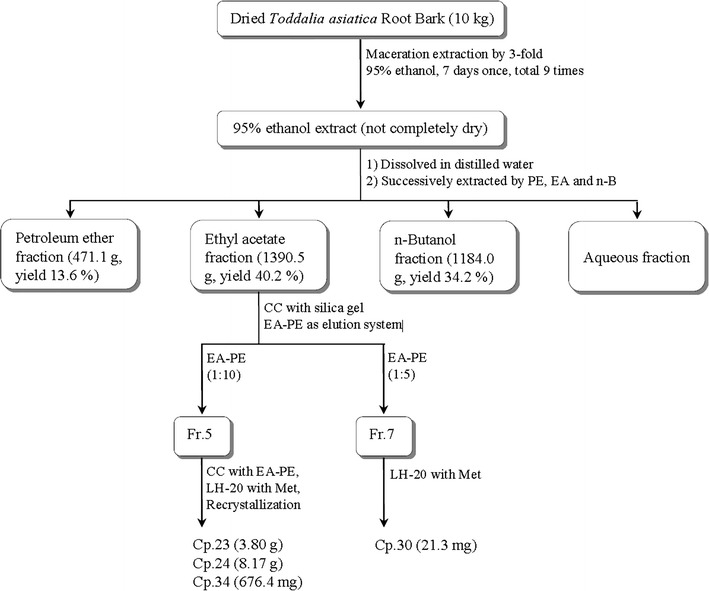
The extraction and fractionation workflow of *Toddalia asiatica* root bark

Ten killogram powder of the dried *T. asiatica* root bark was extracted nine times through maceration method with 95% ethanol in room temperature. All extraction solutions were combined, filtered by Buchner funnel and concentrated in vacuo to yield extract, which was suspended in pure water. Sequential liquid–liquid extraction for successive sample partition was performed by petroleum ether (PE, bp 60–90 °C), ethyl acetate (EA) and *n*-butanol (*n*-B). The extraction and fractionation of *T. asiatica* root bark were according to Fig. 1. For hemostatic activity of all different polarity fractions of *T. asiatica root bark*, bleeding time (BT), amount of bleeding (BA) and clotting time (CT) were as efficacy evaluation indexes by typical mouse tail-cutting method and glass capillary tube method in our previous study [8], average values of BT, BA and CT were (59.67 ± 12. 31) s, (4.42 ± 1.67) mg and (79.67 ± 5.57) s under administration of 1.50 g kg^−1^ ethyl acetate fraction of 95% ethanol extract in Kunming mice, ethyl acetate fraction was found to be the most potential part for further study on hemostatic active substance basis. Systematic separation and purification for main hemostatic natural products from *T. asiatica* root bark were carried out (Fig. 1).

### Chromatographic separation

Chromatographic separation was performed on a Waters ACQUITY UPLC instrument system coupled to a photodiode array detector (PDA), a binary pump, an autosampler and a column compartment (USA), using an Agilent ZORBAX SB-C18 RRHD column (150 mm × 2.1 mm, 1.8 μm). A two-component mobile phase consisting of acetonitrile (A): 0.1% formic acid (B) was used following the elution program: 0 min, A:B (5:95, v/v); 30 min, A:B (90:10, v/v); 40 min, A:B (100:0, v/v). The flow rate was 0.4 mL/min, column temperature was maintained at 30 °C, injection volume was 5 μL by automatic sampling system at 20 °C. An accurately-weighted ethyl-acetate extract was dissolved in absolute methanol (Met), the supernatant was filtered through a 0.22-μm lipophilic microporous filter.

### ESI Q-TOF MS^E^ conditions and data analysis

A Waters Xevo G2-XS Q-TOF (quadrupole time-of-flight) Mass Spectrometer (USA) hyphenated with the Waters ACQUITY UPLC equipped with an ESI source was applied for rapidly identifying the major constituents in ethyl acetate fraction showing hemostatic activity, based on systematic database retrieval. Mass spectrometer and UPLC system were controlled by MassLynx^®^ v4.1 software (Waters, USA). The UPLC effluent after chromatographic separation was introduced into the ESI source without splitting ratio. Data collection was achieved by MS^E^ methodology using two interleaved scan functions with independent collision energies. In this way, a low collision energy scan (Function 1) is immediately followed by a scan in which the collision energy (Function 2) is ramped over a higher range to induce fragmentation of the ions transmitted through the quadruple. This approach enables the simultaneous acquisition of intact precursor ions (protonated molecule [M+H]^+^) and related fragmentions from a single analysis. Each sample was determined in both negative and positive ion modes separately to offer sufficient fragment information.

In this experiment, the related MS parameters were programmed as follows. In MS^E^ centroid section: (1) acquisition: acquisition times, from start time 0 min to end time 40 min; source: ES; acquisition mode, positive or negative polarity, that means each sample was analyzed in positive ion mode and negative ion mode through different sample injections; analyser mode, resolution; dynamic range, extended. (2) TOF MS: Da range, acquire low or high energy over the range of low mass 50 Da to high mass 1000 Da; scan time, 0.5 s; data format, centroid; (3) collision energy: function 1-low collision energy, off; function 2-high collision energy, 40–80 V; (4) cone voltage: override cone voltage value specified in tune file; cone voltage, 40 V. In ES−/ES+ section: source capillary, 2.8 kV; sampling cone, 30; source offset, 60; source temperatures, 120 °C; desolvation temperatures, 450 °C; cone gas flow, 50 L/h; desolvation gas flow, 800 L/h; high-purity helium (He) as collision gas, high-purity nitrogen (N_2_) as nebulizer and auxiliary gas. In LockSpray properties section: acquire LockSpray-apply correction; LockSpray reference compound, 554.2615 Da, leucine-enkephalin (LE); LockSpray acquisition setting: scan time, 0.1 s; interval, 10 s; scans to average, 3; mass windows; 0.5 Da.

## Results and discussion

### Extraction and isolation

After removing the solvent under reduced pressure, 1390.5 g ethyl acetate fraction was subjected to silica gel column chromatography (CC) and was eluted to get fractions 1–11 using a step-gradient solvent system of ethyl acetate (EA):petroleum ether (PE) (0:100 → 100:0). Fr.5 (66.0 g) was further isolated by silica gel CC with EA:PE (1:20 → 1:5) to yield Cp.23 (3.80 g) in EA:PE (1:6), Cp.24 (8.17 g) in EA:PE (1:20) and Cp.34 (676.4 mg) by recrystallization. Fr.7 (5.1 g) was further purified by LH-20 with Met, Cp.30 (21.3 mg) was finally obtained (Fig. 1). Raw spectral analysis data of these four main compounds were listed below.

Isopimpinellin (Cp.23): C_13_H_10_O_5_, yellow crystals. ESI–MS m/z 247.0597 [M+H]^+^, 269.0421 [M+Na]^+^. ^13^C NMR (101 MHz, Chloroform-d): δ 160.62 (C-2), 150.06 (C-7), 145.20 (C-2′), 144.37 (C-5), 143.68 (C-9), 139.58 (C-4), 128.10 (C-8), 114.72 (C-6), 112.75 (C-3), 107.53 (C-10), 105.25 (C-3′), 61.78 (C-8-OCH_3_), 60.84 (C-5-OCH_3_). ^1^H NMR (400 MHz, Chloroform-d): δ 8.06 (d, J = 6.7 Hz, 1H, H-4), 7.58 (d, J = 1.4 Hz, 1H, H-2′), 6.96 (d, J = 1.4 Hz, 1H, H-3′), 6.22 (d, J = 6.7 Hz, 1H, H-3), 4.14 (s, 3H, 8-OCH_3_), 4.10 (s, 3H, 5-OCH_3_). Compared to Ref. [9], isopimpinellin was confirmed.

Pimpinellin (Cp.24): C_13_H_10_O_5_, yellowish needles. ESI–MS m/z 247.0612 [M+H]^+^. ^13^C NMR (101 MHz, Chloroform-d): δ 161.21 (C-2), 150.15 (C-7), 145.73 (C-2′), 144.79 (C-9), 143.54 (C-5), 140.27 (C-4), 135.48 (C-6), 114.47 (C-3), 114.09 (C-8), 109.79 (C-10), 104.67 (C-3′), 62.74 (5-OCH_3_), 61.59 (6-OCH_3_). ^1^H NMR (400 MHz, Chloroform-d): δ 8.09 (d, J = 9.8 Hz, 1H, H-4), 7.66 (d, J = 2.2 Hz, 1H, H-2′), 7.09 (d, J = 2.2 Hz, 1H, H-3′), 6.38 (d, J = 9.8 Hz, 1H, H-3), 4.15 (3H, s, 6-OCH_3_), 4.04 (3H, s, 5-OCH_3_). Compared to Ref. [10], pimpinellin was finally confirmed.

Coumurrayin (Cp.30): C_16_H_18_O_4_, white crystals. ESI–MS m/z 275.1278 [M+H]^+^, 297.1095 [M+Na]^+^. ^13^C NMR (101 MHz, Chloroform-d): δ 161.88 (C-2), 160.90 (C-7), 155.26 (C-5), 153.55 (C-9), 138.93 (C-4), 132.25 (C-3′), 121.84 (C-2′), 110.77 (C-8), 110.16 (C-3), 103.80 (C-10), 90.35 (C-6), 56.06 (C-5-OCH_3_), 55.96 (C-7-OCH_3_), 25.90 (C-5′), 21.46 (C-1′), 17.99 (C-4′). ^1^H NMR (400 MHz, Chloroform-d): δ 7.96 (d, J = 9.6 Hz, 1H, H-4), 6.29 (s, 1H, H-6), 6.12 (d, J = 9.6 Hz, 1H, H-3), 5.18 (d, J = 7.3 Hz, 1H, H-2′), 3.91 (s, 1H, 5-OCH_3_), 3.90 (s, 1H, 7-OCH_3_), 3.41 (d, J = 7.3 Hz, 2H, H-1′), 1.80 (s, 3H, H-4′), 1.64 (s, 3H, H-5′). All data are consistent with Ref. [11], coumurrayin was the identified natural compound.

Phellopterin (Cp.34): C_17_H_16_O_5_, yellowish powder, ESI–MS m/z 301.1422 [M+H]^+^. ^13^C NMR (101 MHz, Chloroform-d): δ 160.59 (C-2), 150.78 (C-7), 145.08 (C-2′), 144.36 (C-5), 144.30 (C-9), 139.72 (C-3″), 139.46 (C-4), 126.79 (C-8), 119.80 (C-2″), 114.45 (C-6), 112.71 (C-3), 107.47 (C-10), 105.10 (C-3′), 70.36 (C-1″), 60.74 (C-5-OCH3), 25.84 (C-3″-CH3), 18.08 (C-3″-CH3). ^1^H NMR (400 MHz, Chloroform-d): δ 8.06 (d, J = 9.7 Hz, 1H, H-4), 7.57 (s, 1H, H-2′), 6.95 (s, 1H, H-3′), 6.22 (d, J = 9.7 Hz, 1H, H-3), 5.55 (d, J = 7.2 Hz, 1H, H-2″), 4.79 (d, J = 7.2 Hz, 2H, H-1″), 4.12 (s, 3H, 5-OCH_3_), 1.68 (s, 3H, 3″–CH_3_), 1.65 (s, 3H, 3″–CH_3_). These are similar to the data of Ref. [12], and phellopterin was confirmed.

### Identification of the constituents in ethyl acetate part by ESI Q-TOF MS^E^

**Fig. 2:**
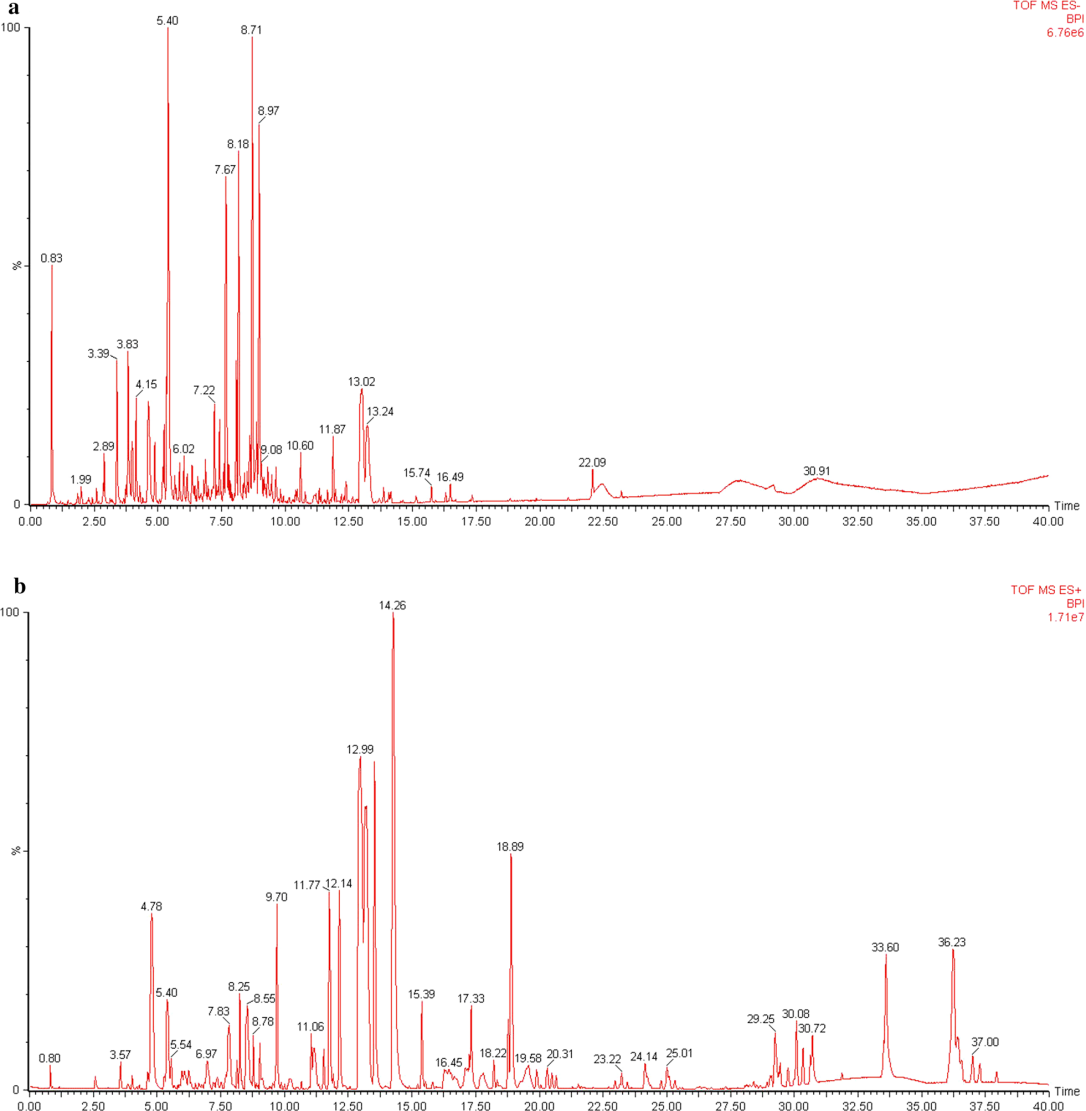
UPLC-ESI-Q-TOF MS^E^ TIC chromatogram of liposoluble extract in *Toddalia asiatica* root bark in negative (−) ion mode (**a**) and positive (+) ion mode (**b**)

**Fig. 3:**
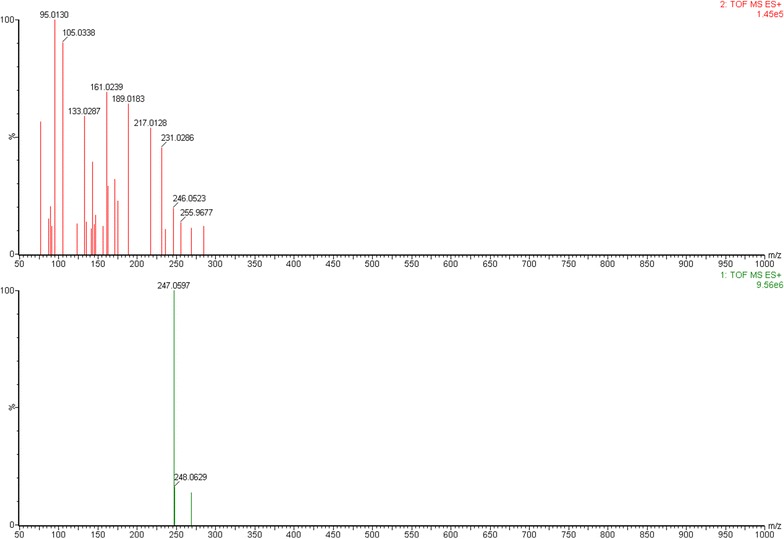
ESI–MS/MS spectra of representative compound isopimpinellin (Cp.23)

**Fig. 4:**
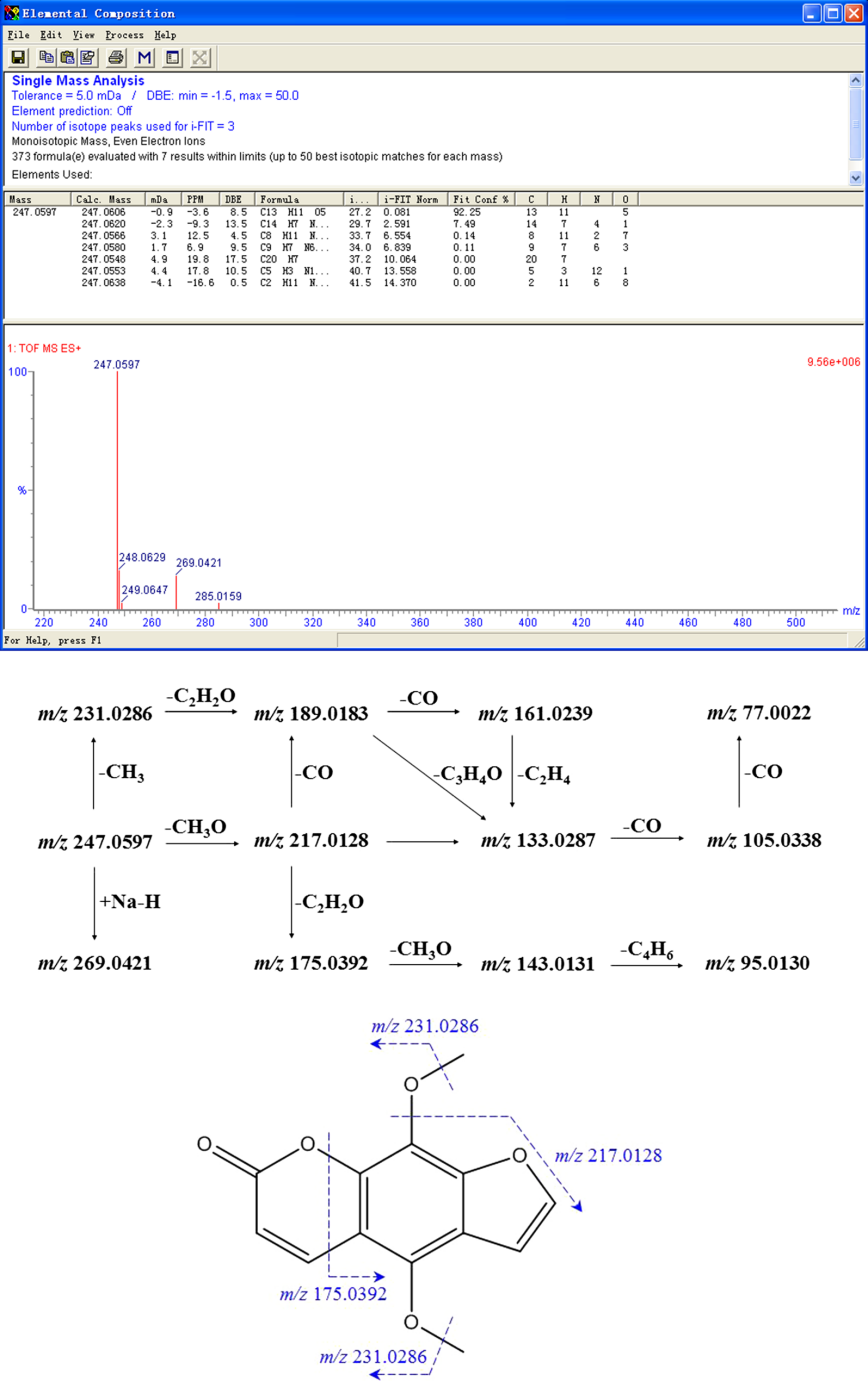
The proposed main fragmentation of representative compound isopimpinellin (Cp.23)

**Fig. 5:**
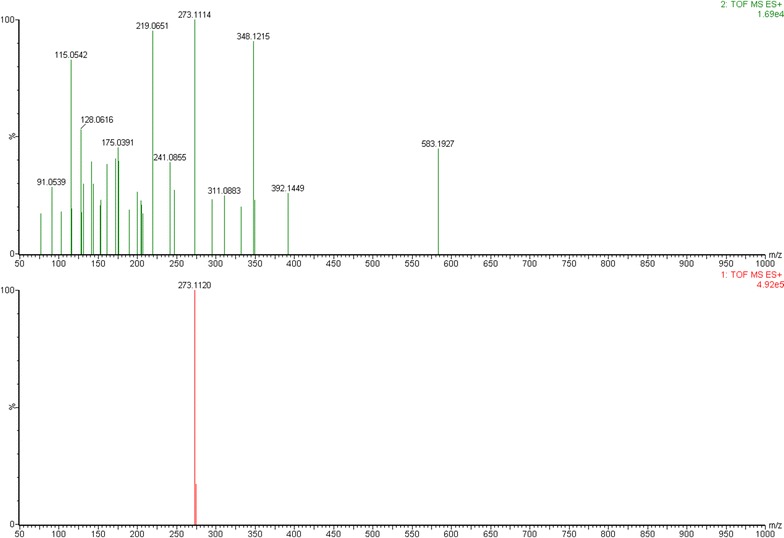
ESI–MS/MS spectra of representative compound dehydrocoumurrayin (Cp.29)

**Fig. 6:**
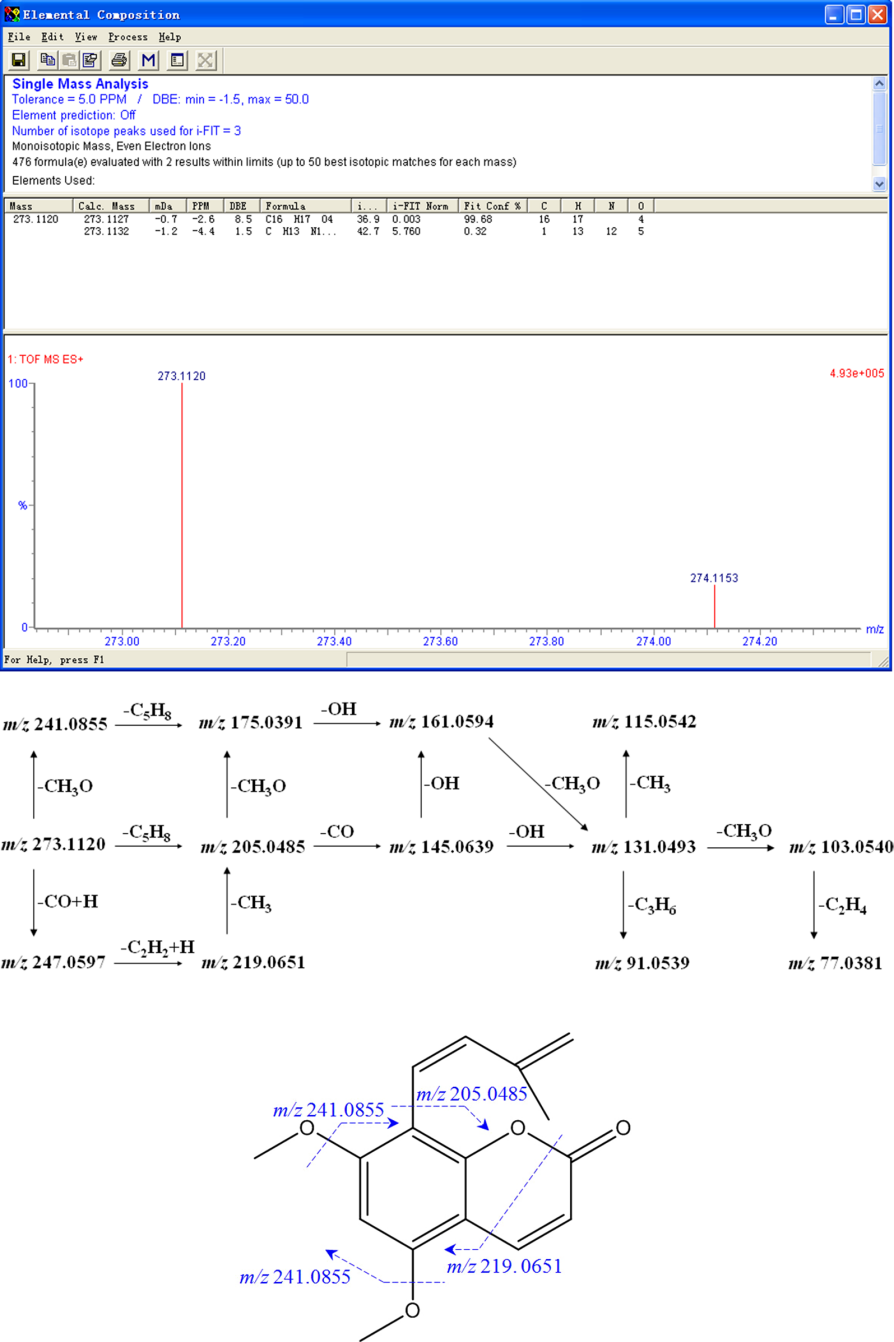
The proposed main fragmentation of representative compound dehydrocoumurrayin (Cp.29)

High-resolution LC–MS/MS analysis was performed to analyze the major constituents in ethyl acetate fraction showing hemostatic activity, all exact molecular weights, all negative and positive ion mode TICs and the secondary daughter ion fragments have been collected by UPLC-ESI Q-TOF MS^E^ detection module (Fig. 2), we totally extracted 47 chromatographic peaks in UPLC-ESI-Q-TOF MS^E^ TIC chromatograms of the liposoluble extract, the deduced molecular formulas from detected exact molecular weights were then easily calculated, commonly accepted <5 ppm threshold and >90 of Fit Conf % of the molecular formulas were finally adopted in this study (Figs. 3, 4, 5, 6). By searching reported known natural products from this herbal medicine in Reaxys database and SIOC chemical database through those deduced molecular formulas and detected exact molecular weights (Table 1), tentative identification of the constituents in ethyl acetate part of *T. asiatica* was done firstly. Then the conclusive research reports in the literature about this herbal medicine in CNKI [13] were systematically found out by searching target molecular formulas and this herbal medicine name *T. asiatica*, 飞龙掌血 or Feilong Zhangxue. The preliminarily deduced compounds were further screened and confirmed. After that, feasible fragmentation pathways of the very-likely candidate compounds from *T. asiatica* and their chemical structures were all analyzed and withstood the scientific scrutiny according to the tested negative or positive mode MS^E^ information, the usual fragmentation pathways contained –CH_3_, –OCH_3_, –OH, –CO, –C_2_H_4_, etc. The differences of chromatographic retention behavior of similar natural compounds were also applied to ensure the correct results between those easily confused compounds.

**Table 1:** Identified natural compounds of *Toddalia asiatica* root bark by UPLC-QTOF-MS/MS

No.	Rt (min)	Molecular formula	Molecular weight	−MS^2^ [parent ion m/z]: daughter ion	+MS^2^ [parent ion m/z]: daughter ion	Compound identification	Reaxys no.	SIOC SRN	CAS no.	References
1	0.826	C_12_H_22_O_11_	342.2964	[M−H 341.1078, M+Cl 377.0841, M−H+FA 387.1133]: 89.0233, 113.0232, 161.0442, 179.0546, 212.0526, 341.1071, 377.0841	–	Maltose	93798	24426146	69-79-4	–
2	2.894	C_15_H_16_O_9_	340.2821	[M−H 339.0711, M+Cl 375.0686]: 89.0231, 94.9239, 121.0282, 146.9818, 162.8379, 174.0010, 339.0715, 375.0650	–	Esculin	95387	60113315	531-75-9	–
3	3.389	C_15_H_16_O_9_	340.2821	[M−H 339.0710]: 89.0236, 112.9840, 146.9816, 174.0009, 219.0287, 249.0393, 339.0710	[M+H 341.0860, M+Na 363.0674, M+K 379.0404]: 77.0392, 137.0227, 147.0445, 163.0394, 191.0338, 258.9890, 341.0860, 365.1159, 379.0418	Daphnin	49440	60108006	486-55-5	–
4	4.623	C_20_H_23_NO_4_	341.4009	[M−H 340.1547]: 89.0237, 196.0516, 201.0370, 224.0467, 252.0417, 310.1077, 340.1544, 341.1578	[M+H 342.1704]: 152.0627, 165.0706, 178.0779, 189.0706, 193.0655, 222.0679, 342.1704, 343.1738	–	–	–	–	Unknown
5	5.190	C_22_H_38_O_13_	510.5293	[M−H 509.2231, M−3H+FA 553.2493]: 71.0132, 89.0236, 146.9822, 165.0540, 185.0050, 201.0370, 553.2500	–	[R-(E)]-1-[8-(β-d-Glucopyranosyloxy)-2,6-dimethyl-2-octenoate] β-d-glucopyranose	6944877	250327357	158921-22-3	–
6	5.401	C_18_H_19_NO_4_	313.3478	–	[M+H 314.1756]: 77.0387, 107.0493, 121.0652, 165.0701, 177.0550, 194.0726, 314.1756	*N*-cis-Feruloyl tyramine	5482514	–	65646-26-6	–
7	5.543	C_22_H_24_O_5_	368.4229	[M−H 367.1027, M−2H+Na 389.0848]: 93.0338, 134.0362, 173.0445, 191.0552, 367.1027, 389.0848	[M+H 369.1168, M+Na 391.0987]: 89.0384, 145.0286, 177.0547, 214.0892, 314.1745, 369.1168, 391.0987	5-Methoxy-8-geranyloxypsoralen	7101938	246688070	17182-52-4	[6, 14]
8	5.844	C_32_H_38_O_10_	582.6381	[M−H 581.2228, M−H+FA 627.2288]: 89.0237, 146.9823, 174.0011, 201.0370, 261.0766, 359.1128, 513.1600, 581.2236	–	Toddalin B	26980667	–	1538606-91-5	–
9	6.253	C_19_H_21_NO_4_	327.3743	–	[M+H 328.1908]: 77.0390, 115.0548, 121.0654, 156.0420, 165.0711, 254.0551, 328.1908	(S)-Isocoreximine	1552902	–	14140-19-3	[15]
10	7.418	C_19_H_24_O_11_	428.3872	[M−H 427.1245]: 119.0494, 145.0290, 176.0108, 191.0352, 235.0246, 250.0480, 265.0717, 367.1395, 427.1245	–	3-(4,7-Dimethoxy-6-((2S,3R,4S,5S,6R)-3,4,5-trihydroxy-6-(hydroxymethyl)-tetrahydro-2H-pyran-2-yloxy)benzofuran-5-yl) propanoic acid	–	55840374	169312-05-4	–
11	7.685	C_28_H_34_O_15_	610.5605	[M−H 609.1814]: 151.0025, 164.0105, 242.0572, 286.0471, 323.0525, 609.1819	[M+H 611.1978, M+Na 633.1788, M+Ka 649.1527]: 85.0286, 153.0186, 177.0546, 303.0867, 633.1780, 649.1512	Hesperidin	75140	60111601	520-26-3	[16]
12	8.145	C_16_H_20_O_6_	308.3264	–	[M+H 309.1345]: 91.0546, 147.0445, 177.0547, 205.0497, 219.0655, 291.1227, 309.1336	Mexoticin	–	1027046	18196-00-4	[15, 17]
13	9.697	C_16_H_20_O_6_	308.3264	–	[M+H 309.1342, M+Na 331.1158, 2M+Na 639.2419]: 91.0544, 119.0496, 131.0496, 147.0443, 177.0548, 205.0501, 219.0653, 309.1328, 331.1134, 347.0866, 639.2419	Toddalolactone	5445192	80582	483-90-9	[15, 17]
14	10.200	C_21_H_18_NO_4_	348.3719	–	[M+H 349.1261]: 91.0545, 127.0398, 147.0447, 177.0555, 232.0762, 246.0914, 274.0868, 290.0819, 304.0977, 332.0928	Chelerythrine	3915260	60513600	34316-15-9	–
15	10.600	C_32_H_36_O_14_	644.6198	[M−H 643.2034]: 89.0239, 135.0442, 145.0287, 191.0553, 367.1393, 409.1494	[M+H 645.2172, M+Na 667.1964]: 91.0545, 112.8961, 147.0448, 177.0554, 246.0774, 291.1245, 475.1619, 679.2251	Methyl 2,3-dibenzoyl-4-*O*-(2,3,4-tri-*O*-acetyl-α-l-rhamnopyranosyl)-α-d-xylopyranoside	–	24368097	99104-82-2	–
16	11.060	C_15_H_16_O_5_	276.2845	**–**	[M+H 277.1071, M+Na 299.0925, 2M+Na 575.1879]: 91.0542, 112.8963, 147.0444, 177.0546, 299.0888, 315.0627, 575.1851	Toddanin	–	–	213483-74-0	[15]
17	11.538	C_12_H_12_O_5_	236.2207	–	[M+H 237.0759, M+Na 259.0576]: 77.0385, 112.8953, 156.0428, 177.0546, 207.0651, 275.0294	5,7,8-Trimethoxycoumarin	1348912	3321716	60796-65-8	[14, 15]
18	11.765	C_14_H_13_NO_4_	259.2573	–	[M+H 260.0924]: 77.0385, 128.0496, 156.0445, 184.0392, 202.0497, 219.0650	Skimmianine	28904	60075401	83-95-4	[19]
19	11.907	C_33_H_38_O_14_	658.6464	[M−H 657.2189, M−H+FA 703.2241]: 89.0235, 119.9463, 134.0366, 146.9650, 174.0011, 193.0498, 367.1395	[M+H 659.2340, M+Na 681.2154]: 89.0384, 117.0339, 145.0289, 161.0601, 177.0549, 219.0655, 320.0914, 681.2154, 697.1884	Toddalin A	26980663	–	1538606-87-9	[18]
20	12.157	C_13_H_11_NO_3_	229.2314	–	[M+H 230.0829]: 89.0392, 116.0504, 144.0452, 172.0409, 200.0356	Gamma-fagarine	212820	60112081	524-15-2	–
21	12.970	C_36_H_39_NO_8_	613.6968	[M−H 612.2595, M−H+FA 658.2648]: 89.0233, 112.9845, 160.0153, 175.0388, 201.0367, 253.0490, 311.1149, 326.1388, 376.1546, 509.1229, 582.2126, 612.2589	[M+H 614.2753]: 133.0652, 161.0602, 205.0499, 219.0656, 243.0662, 273.1118, 294.0889, 309.1115, 463.1184, 513.1536, 614.2748	–	–	–	–	Unknown
22	13.215	C_36_H_39_NO_8_	613.6968	[M−H 612.2587, M−H+FA 658.2639]: 89.0232, 112.9844, 160.0151, 174.0003, 201.0360, 225.0538, 311.1143, 326.1378, 376.1529, 509.1223, 582.2113, 612.2576	[M+H 614.2740]: 105.0701, 133.0651, 161.0598, 205.0497, 219.0652, 243.0658, 273.1111, 294.0883, 309.1109, 463.1169, 614.2737	–	–	–	–	Unknown
23	13.516	C_13_H_10_O_5_	246.2155	–	[M+H 247.0597, M+Na 269.0421]: 77.0022, 95.0130, 105.0338, 133.0287, 161.0239, 175.0392, 189.0183, 217.0128, 231.0286, 269.0420, 285.0153	Isopimpinellin	262337	60107409	482-27-9	[20, 21]
24	14.311	C_13_H_10_O_5_	246.2155	–	[M+H 247.0612]: 77.0392, 91.0549, 105.0345, 133.3947, 147.0452, 161.0242, 175.0398, 217.0148, 231.0300, 246.0536	Pimpinellin	241751	43394	131-12-4	[20, 21]
25	15.365	C_20_H_18_NO_4_	336.3612	–	[M+H 337.1642, M+Na 359.1459, 2M+Na 695.3019]: 91.0544, 131.0495, 147.0444, 205.0499, 219.0657, 359.1465, 375.1199, 695.3039	Berberine	3570374	262538	2086-83-1	–
26	16.930	C_21_H_17_NO_5_	363.3634	–	[M+H 364.1166, M+K 402.3748]: 112.8966, 156.0426, 219.0656, 273.1121, 348.1220, 363.1085, 364.1166	Oxynitidine	345213	60114967	548-31-2	[15, 19]
27	17.250	C_21_H_17_NO_5_	363.3634	–	[M+H 364.1174, M+Na 386.0988]: 177.0575, 219.0652, 273.1118, 291.0523, 306.0756, 386.0988	Oxychelerythrine	345198	1471260	28342-33-8	[15, 19]
28	17.334	C_11_H_10_O_5_	222.1941	–	[M+H 223.0599, M+Na 244.0343]: 119.0131, 147.0079, 165.0186, 244.0343	8-Hydroxy-5,7-dimethoxycoumarin	1348197	3833130	61899-44-3	[14]
29	18.186	C_16_H_16_O_4_	272.2958	–	[M+H 273.1120, M+2K 348.1215]: 77.0381, 91.0539, 103.0540, 115.0542, 131.0493, 145.0639, 161.0594, 175.0391, 219.0651, 241.0855, 247.0597, 273.1120	Dehydrocoumurrayin	7712465	250818025	178275-73-5	[14, 18]
30	18.775	C_16_H_18_O_4_	274.3117	–	[M+H 275.1278, M+Na 297.1095]: 77.0383, 91.0540, 103.0542, 131.0494, 147.0440, 156.0426, 175.0392, 204.0417, 275.1291	Coumurrayin	1291821	951608	17245-25-9	[18]
31	19.579	C_16_H_16_O_4_	272.2958	–	[M+H 273.1127, M+Na 295.0946]: 115.0542, 161.0598, 219.0651, 273.1115, 304.0960, 332.0907, 348.1220	5,7-Dimethoxy-8-(3′-methylbuta-1,3′-dienyl) coumarin	3555720	248514399	106940-77-6	[18]
32	20.254	C_16_H_16_O_4_	272.2958	–	[M+H 273.1120]: 91.0542, 115.0547, 161.0596, 219.0651, 241.0855, 273.1119, 304.0966, 332.0911, 348.1220	6-(3-Methyl-1,3-butadienyl)-5,7-dimethoxycoumarin	27076142	–	–	[14]
33	20.306	C_16_H_18_O_4_	274.3117	–	[M+H 275.1277, M+Na 297.1095]: 90.0463, 118.0418, 161.0601, 273.1121, 275.1275, 297.1100	Toddaculine	1291825	417827	4335-12-0	[18]
34	23.153	C_17_H_16_O_5_	300.3059	–	[M+H 301.1422]: 81.0701, 114.0916, 149.0240, 156.0424, 204.0416, 217.0520, 233.0739, 273.1122, 301.1412	Phellopterin	297173	60176623	2543-94-4	[14, 15]

Here we took Cp.23, Cp.29 as examples to clarify the identification process of proposed practical strategies in this study. Cp.23 was separated as a peak in t_R_ 13.516 min its positive ion mode TICs and the secondary daughter ion fragments have been collected by UPLC-ESI Q-TOF MS^E^ detection module, but only in positive ion mode TIC showed obvious MS signals in unit of e6. M+H 247.0597, M+Na 269.0421 and M+K 285.0159 were easily found as quasi-molecular ion peak, adduct ion peak in 1 TOF MS ES^+^. C_13_H_10_O_5_ could be calculated out by ‘Elemental Composition’ analytical tool, with mass error −3.6 ppm, −0.9 mDa and calc. mass 247.0606 [M+H]^+^. In Reaxys and SIOC chemical database, there were only two compounds obtaining the same molecular formula C_13_H_10_O_5_. Then in relation to 2 TOF MS ES^+^ of Cp.23, several fragment ion peaks could help me to presumably infer the possible lost fragment ions and chemical structures, Figs. 3 and 4 showed the proposed main fragmentation of Cp.23. The deprotonated precursor ion (m/z 247.0597) could be fragmented into m/z 231.0286 ([M+H–CH_3_]^+^), 217.0128 ([M+H–CH_3_O]^+^). m/z 231.0286 was forced to lose [–C_2_H_2_O] under electron bombardment or collision, and produced m/z 189.0183, then m/z 161.0239 without [–CO], m/z 133.0287 drawing off [–C_2_H_4_]. Relatively small fragment ions also could be obtained, such as m/z 105.0338, m/z 77.0022. m/z 217.0128 was not only split up into above-referenced fragment ions through losing [–CO], [–C_3_H_4_O], et al., but also divided to m/z 175.0392, m/z 143.0131, m/z 95.0130 by dissociating [–C_2_H_2_O], [–CH_3_O] and [–C_4_H_6_]. After the relevant research literature and reference substance comparison, Cp.23 was inferred as isopimpinellin.

The peak of Cp.29 in t_R_ 18.186 min had a molecular formula of C_16_H_16_O_4_ (m/z 273.1120 for [C_16_H_17_O_4_]^+^, mass error −2.6 ppm). The deprotonated precursor ion (m/z 273.1120) was fragmented into m/z 247.0597 ([M+H–CO]^+^), 241.0855 ([M+H−CH_3_O]^+^) and 205.0485 ([M+H–C_5_H_8_]^+^). Other fragments m/z 175.0391, 161.0594, 131.0493, 115.0542, 103.0540 could be easily deduced through MS conventional cleavage, such as –CO, –OH, –CH_3_O (Figs. 5, 6). Reaxys database showed three natural compounds with the same molecular formula (C_16_H_16_O_4_) from *T. asiatica* (Table 1), dehydrocoumurrayin was finally confirmed through the literature reference, the polarity difference between the compounds.

**Fig. 7:**
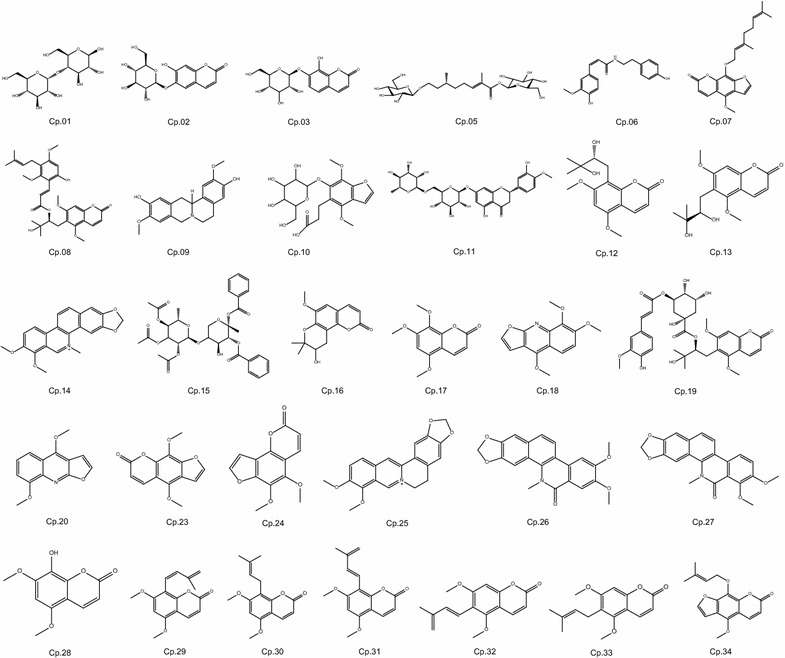
Natural compounds from *Toddalia asiatica* root bark in this study

Other natural products were identified following the same manner. Isopimpinellin (Cp.23), pimpinellin (Cp.24), coumurrayin (Cp.30), phellopterin (Cp.34) as main isolated compounds in this part of *T. asiatica* root bark through our natural product extraction and separation were also detected and verified through these strategies. As a result, a total of 31 natural compounds in *T. asiatica* root bark got identified or putatively characterized based on above-mentioned strategies (Figs. 7).

## Conclusion

In this report, hemostatic chemical constituents from natural medicine *T. asiatica* root bark were first investigated by LC-ESI Q-TOF MS^E^ and bioassay-guided compounds’ extraction and isolation from this natural medicine. There were totally 31 natural compounds in *T. asiatica* root bark which got putatively characterized, four main coumarins were purified and identified. These findings would show us more clear material basis of this complex medicine, guiding further pharmaceutical research of *T. asiatica* efficiently and comprehensively.

Our study illustrated that the sensitive UPLC–Q-TOF analytical system combined with the MS^E^ method of fragmentation data collection and natural product databases, allows a relatively rapid and reasonable investigation of the reported and unknown compounds in this complex TCM sample *T. asiatica*. This proposed method could be a candidate strategy to study TCM or other complex natural medicines so far in facing current bottleneck situation of natural medicine development, this would better promote development and application of natural medicines and their medicinal resources.

## Abbreviations

CAS: Chinese Academy of Sciences
CC: column chromatography
CNKI: China national knowledge infrastructure
PE: petroleum ether
EA: ethyl acetate
FA: formic acid
n-B: *n*-butanol
Cp.: compound
Met: methanol
DCM: dichloromethane
TCM: traditional Chinese medicine
